# Urea Cycle Defects: Early-Onset Disease Associated with A208T Mutation in OTC Gene—Expanding the Clinical Phenotype

**DOI:** 10.1155/2017/1048717

**Published:** 2017-02-05

**Authors:** Ana Isabel Sánchez, Alejandra Rincón, Mary García, Fernando Suárez-Obando

**Affiliations:** ^1^Instituto de Genética Humana, Facultad de Medicina, Pontificia Universidad Javeriana, Bogotá D.C., Colombia; ^2^Instituto de Genética Humana, Facultad de Medicina, Pontificia Universidad Javeriana, Hospital Universitario San Ignacio, Bogotá D.C., Colombia

## Abstract

Ornithine transcarbamylase deficiency (OMIM: 311250) is the most common disorder of urea cycle disorders, accounting for nearly 50% of all cases. We report a case of a two-month- old male patient, who attends our medical genetics consultation because of low citrulline levels and elevated glutamine to citrulline ratio detected by expanded newborn screening with tandem mass spectrometry. He is an asymptomatic male with a normal physical examination and appropriate neurodevelopmental milestones. The patient has a family history of one older brother who died at 18 months old from severe and sudden hyperammonemia and a maternal aunt who suddenly died at two years old. He had high plasma ammonium concentration and a confirmed OTC mutation (p.A208T). Usually, this mutation causes OTC deficiency of late onset in adult males. However, this report raises awareness about mutations previously described as a late-onset causing disease, which can cause severe hyperammonemia and high risk of dying at an early age.

## 1. Introduction

Urea cycle disorders (UCD) comprise at least eight conditions, caused by a deficiency of one of the essential proteins for the metabolism of nitrogen [1]. Varying degrees of deficiency or total absence of activity of any of these enzymes ends up in the accumulation of ammonium and other metabolites that are toxic within few hours or days after birth [2]. Hyperammonemia leads to severe central nervous system dysfunction [3] (Gropman et al. 2007) by altering amino acid and neurotransmitters pathways and by interfering with normal cerebral energy metabolism and oxidative stress [2]. Ammonium toxicity to the brain provokes irreversible damage due to cortical atrophy, edema, and demyelination, resulting in seizures, coma, and even death [2]. Despite aggressive treatment, survivors may develop intellectual disability, which is directly related to the frequency, severity, and duration of the hyperammonemia episodes [4].

Ornithine transcarbamylase (OTC) deficiency (OMIM: 311250) is the most frequent cause of UCD [5–7], accounting for nearly 50% of all cases [8]. It is an X-linked disorder, in which hemizygous males are almost always symptomatic and about 20% of female carriers, may present some neurocognitive deficit depending on lyonization patterns [9, 10].

The OTC is a mitochondrial enzyme that catalyzes the reaction between carbamoyl phosphate and ornithine to form citrulline, an essential step for the formation of nontoxic urea from ammonium [8]. The clinical symptoms of OTC deficiency are a consequence of the toxic effects of ammonium and include recurrent vomiting, lethargy, hyporexia, neurobehavioral changes, and seizures. Biochemical testing shows the elevation of plasma glutamine and elevated levels of urinary orotic acid.

The OTC gene (Xp21.1) contains ten exons and nine introns which codifies a protein of 322 amino acids (36.1 kDa) expressed in the liver and the intestinal mucosa [9, 11, 12]. There are approximately 417 disease-causing mutations in the OTC gene [4]. The majority of them are missense, resulting in amino acid substitution and therefore affecting the function or structure of the enzyme [9]. The phenotype ranges from acute neonatal hyperammonemia coma or neonatal death to asymptomatic adults [13].

There is a correlation between clinical phenotype and genotype. Almost all patients who present within the first days of life with acute hyperammonemia coma, or having peak ammonium levels of more than 1000 *μ*M, do have mutations that completely abolish enzymatic activity, in contrast to those patients with late onset and a milder phenotype, who have mutations that allow some residual enzymatic activity. Thus, some mutations found in patients with OTC deficiency are associated with early onset presentation and severe disease, in contrast to some patients that are related to late-onset disease or have an asymptomatic condition [4, 6].

We report a case of OTC deficiency in a patient with early-onset hyperammonemia, with a family history of a brother who died at 18 months of age because of severe hyperammonemia, rapid neurological deterioration, and brain death. Our patient carried a specific mutation previously reported as a late-onset cause of mild hyperammonemia.

## 2. Case Presentation

We present the case of an asymptomatic male who was brought to our outpatient clinic at two months old because of low citrulline levels and high glutamine-to-citrulline ratio, detected by expanded newborn screening with tandem mass spectrometry. He was born at thirty-nine weeks of gestation by cesarean section, after an uneventful pregnancy from two healthy nonconsanguineous parents. Birth weight was 3.510 g and length was 51 cm. The patient was in good general conditions; physical examination and neurodevelopmental milestones were normal by his age. Family history was significant in the patient: he had one older brother who died at 18 months old from severe and sudden hyperammonemia leading to neurological deterioration, respiratory alkalosis, and brain death. He was born after a full-term uneventful gestation, by cesarean section. Birth weight was 3600 grams and length was 56 cms. He received exclusive breastfeeding for three months, after that he was placed on milk formula until he was six months old when complementary feeding was started. He was healthy until he was 18 months old when he started having continuous vomiting during three days, poor feeding, and deterioration of consciousness. Ammonia levels were found to be suddenly high. He deteriorated rapidly afterward and died three days later. A medical autopsy was done, without any significant findings. No further molecular studies were performed. Family history was also significant for the sudden death of a maternal aunt at two years old.

Biochemical analysis in the patient revealed hyperammonemia (138.1 *μ*mol/L, normal value: 27.2–102 *μ*mol/L), which was persistent, with partially compensated respiratory alkalosis. High-performance liquid chromatography (HPLC) measurement of serum amino acids revealed high levels of glycine (559 mmol/L; normal value up to 426 *μ*mol/L), alanine (1090 *μ*mol/L; normal value up to 474 *μ*mol/L), ornithine (132 *μ*mol/L; normal value up to 130 *μ*mol/L), and glutamine (1464 *μ*mol/L; normal value up to 426 *μ*mol/L) and revealed lower plasma levels of citrulline (4 *μ*mol/L; normal value: 9–38) and a higher urine orotic acid at 4.7 mmol/mol and creatinine (normal value: 1.0–3.2 mmol/mol creatinine).

The biochemical profile was highly suspicious of an ornithine transcarbamylase defect. Clotting studies, liver enzymes, and renal function tests were normal. We performed an NGS-based panel testing of eight genes (ALDH18A1, ARG1, ASL, ASS1, CPS1, HMGCL, OAT, and OTC) revealing a hemizygous mutation in exon 6 of OTC gene (c.622G>A), which determines a substitution of alanine by threonine in the protein (p.A208T). The patient was placed on regular outpatient follow-up and treated with low protein diet, achieving a good metabolic control.

## 3. Discussion

OTC deficiency is the most common inherited disease of ureagenesis with an estimated prevalence of 1 in 62,000 to 1 in 77,000 [8, 14, 15]. Among all cases of severe hyperammonemia, OTC deficiency should always be considered as a differential diagnosis. The classical form with neonatal onset, severe hyperammonemia, and coma are well recognized as the most common presentation of the disease; however, some studies have reported a smaller proportion of individuals with neonatal onset of OTC deficiency than those with onset after the neonatal period [1].

To date, approximately 417 disease-causing mutations have been reported in the OTC gene [4]. Most of them are single-base pair substitutions causing missense or nonsense mutations, whereas a smaller proportion is due to deletions, insertions, splice site mutations, and regulatory mutations [16]. Many authors have reported several OTC mutations causing late-onset or early-onset disease [8, 17].

Our patient has a mutation (A208T), previously reported as a mutation causing late-onset disease in adult males since this mutation preserves the residual enzymatic activity of about 4% (Ausems et al., 1997; Tuchman et al., 1994; Cavicchi et al., 2014). It is noteworthy to mention that the patient's brother died because of coma and hyperammonemia at infancy, suggesting no residual activity of the enzyme. On the other hand, our patient presented high ammonium levels at an early age, being himself asymptomatic.

Although A208T mutation is commonly described in men with the late-onset disease, it was reported in a family with three patients who died at four months old [18]. Here we describe a patient with persistent but asymptomatic hyperammonemia, with a significant family history of a brother, who died at an early age because of severe hyperammonemia and who probably had the same mutation in the OTC gene.

It is also noteworthy to mention that his aunt presented sudden death at two years old with no explanation, which retrospectively may be compatible with OTC deficiency. Females who are heterozygous may be symptomatic in adulthood under metabolic stress [19], because of different patterns of skewed X-inactivation. However, she died in infancy with no obvious triggered condition like an infection or any other metabolic stress. Also, it has to be said that specifically A208T mutation has been reported previously in heterozygous asymptomatic females [18].

This report raises awareness that mutations previously described in the literature as a late-onset causing disease may be found in patients with severe hyperammonemia with a high risk of dying at an early age.
